# Association of ST-T changes with all-cause mortality among patients with peripheral T-cell lymphomas

**DOI:** 10.1515/med-2022-0517

**Published:** 2022-07-12

**Authors:** Hanzhi Du, Lihong Yang, Bin Yan, Juan Zhao, Mengchang Wang

**Affiliations:** Department of Haematology, The First Affiliated Hospital of Xi’an Jiaotong University, Xi’an, China; Department of Clinical Research Centre, The First Affiliated Hospital of Xi’an Jiaotong University, Xi’an, China

**Keywords:** electrocardiogram parameters, lymphomas, prognosis

## Abstract

Research has suggested a significant prognostic value of ST-T changes in various cardiovascular diseases and malignant tumors. However, their role in predicting prognosis in patients with peripheral T-cell lymphomas (PTCLs) remains unknown. Here, we investigated the prognostic potential of ST-T changes in all-cause mortality of PTCLs patients. In total, 131 patients with PTCLs between January 2015 and April 2020 were enrolled. Univariable and multivariable COX proportional hazards regression models were used to find the relationship between ST-T changes and all-cause mortality in these patients. A significant difference in all-cause mortality was found between patients with ST-T abnormalities and those without definite abnormalities in the ST-T segments (*P* = 0.027). Multivariable Cox risk regression analysis indicated that patients with ST-T changes had greater all-cause mortality than patients with normal ST-T segments in the intermediate-high/high-risk groups (*P* < 0.001). In addition, ST-T changes were markedly distinction in patients with hypoproteinemia than in those with no definite abnormalities in the ST-T segments (*P* = 0.021). ST-T changes may serve as potential, simple, and effective prognostic factors for all-cause mortality in PTCLs patients, especially in the intermediate-high/high-risk and hypoproteinemia groups. Therefore, regular ECG monitoring is recommended to guide the clinical treatment of patients with PTCLs.

## 1 Introduction

Peripheral T-cell lymphomas (PTCLs) are a nonuniform group of diseases that originate in mature postthymic T lymphocytes. They represent about 10–15% of non-Hodgkin’s lymphomas in European and American countries, and approximately 21.4% in China [1,2]. PTCLs are more frequent in men and older adults. Although clinical manifestations may vary greatly depending on the various subtypes and stages, the prognosis is usually poor. In some PTCL subtypes, the 5-year survival rate may be as low as 7%. Patients with intermediate-high/high-risk are often at a greater risk for poor prognosis [3,4,5].

Anthracycline-based regimens remain the cornerstone of treatment for PTCLs [6,7,8,9]. Other second-line anticancer chemotherapeutics are recommended for relapsed and refractory patients, including antimetabolites 5-fluoropyrimidine, cytarabine, alkylating agents, platinums, monoclonal antibodies, and antimicrotubule drugs. The overall response rates are 50–60%, and the complete remission rates are 20–30% [10]. However, there is an increased risk of cardiovascular adverse events in patients receiving treatment. These drugs can directly or indirectly cause certain cardiotoxicity, mainly manifested as arrhythmia, dilated cardiomyopathy, ventricular dysfunction, heart failure, myocardial infarction, pericarditis, and pericardial effusion [6,11,12]. Consequently, it is important to evaluate the cardiac burden and optimize appropriate treatment strategies to improve outcomes and patient survival.

The 12-lead electrocardiogram (ECG) is a simple, noninvasive, and inexpensive tool in clinical practice. ST-T represents the electrical activity from ventricular contraction to ventricular relaxation. The manifestation of the ST segment and T wave on ECG beyond the normal range is called ST-T changes. Recently, ST-T changes were demonstrated as strong prognostic indicators of coronary artery disease, acute myocarditis, systemic sclerosis (scleroderma), and various types of malignant tumors, including gastrointestinal tumors and breast cancer [13,14,15,16,17,18]. Nevertheless, there is no evidence indicating an exact relationship between ST-T changes and PTCLs prognosis. During this study, we explored the potential predictive role of ST-T changes in mortality from all-cause among PTCL patients.

## 2 Patients and methods

### 2.1 Patients

From January 2015 and April 2020, 131 patients diagnosed with PTCLs in the First Affiliated Hospital of Xi’an JiaoTong University were incorporated into this study. Diagnosis, subtypes, and treatment were assigned according to the 2016 National Comprehensive Cancer Network Peripheral T-cell Lymphoma Clinical Practice Guidelines [19]. Patients with congenital heart disease, acute coronary syndrome, atrial fibrillation, and other arrhythmias, and lack of follow-up information were excluded. The present study obtained the approval by the Medical Ethics Review Committee of the First Affiliated Hospital of Xi’an JiaoTong University (NO. XJTU1AF2020LSK-179).

### 2.2 Clinical characteristics

Clinical baseline characteristics and demographics of PTCL patients were extracted from the patient’s electronic medical records based on the inclusion and exclusion criteria. Clinical staging referred to the Ann Arbor classification revised by 2014 Lugano evaluation standard [20]. Eastern Cooperative Oncology Group (ECOG) scoring criteria were defined as an indicator of the general health status and tolerance to treatment from physical activity in patients, and the scores ranged from 0 to 5. The International Prognostic Index (IPI) score system, which is the most widely accepted and used clinical prognostic scoring system [21], was utilized for non-Hodgkin’s lymphoma and included five prognostic indicators: age, serum lactate dehydrogenase (LDH), ECOG score, clinical stage, and the number of involved extranodal invasion sites. Furthermore, the IPI was calculated for patient risk stratification into the following categories: IPI score 0–1 as low-risk, 2 as low-intermediate risk, 3 as intermediate-high risk, and 4–5 as high-risk. Given the relatively small sample size and other factors, we divided the groups into two categories (low/low-intermediate risk and intermediate-high/high-risk). Moreover, patients were categorized into the normal (≥3.5 g/dL) and hypoproteinemia (<3.5 g/dL) groups. All-cause mortality was defined as the endpoint, and the last follow-up was July 31, 2020. Follow-up was conducted by telephone or outpatient visits. ST-T changes in PTCL patients were evaluated. ECG reports were analyzed by two professional technicians in the ECG room of the First Affiliated Hospital of Xi ‘an Jiaotong University independently, and any disagreement was discussed till an unanimous decision was made.

### 2.3 Statistical analysis

Continuous data are presented as mean ± SD or median (interquartile range), and categorical data are presented as percentage (*n*%). The normality and homogeneity of the variable distributions were determined using the Shapiro Wilks test and Levene’s test, respectively. Survival curves were built by the Kaplan–Meier method, and survival differences were tested by the log-rank test. The association between ST-T changes and mortality from all-cause was performed using univariable and multivariable Cox proportional hazard models. Covariates for the multivariable Cox proportional hazard regression models were selected as potential confounders based on *P* < 0.05 or their biological relevance in univariable analyses. Assumptions for Cox proportional hazards regression were tested and met with the Schoenfeld residuals. Hazard ratios (HRs) and 95% confidence intervals (CIs) were estimated using Cox proportional hazards regression, adjusting for potential confounders and covariates. Statistical significance was set at *P* < 0.05. All statistical analyses were conducted using SPSS version 26.0 (IBM Corp., Armonk, NY).

## 3 Results

### 3.1 Patient characteristics

In total, 131 patients with PTCLs, and complete electronic medical records were analyzed retrospectively. The clinical baseline characteristics of PTCLs patients are listed in Table 1. In this study, 84 (64.1%) men and 47 (35.9%) women with the average age of 50.6 ± 18.3 years were comprised. The mean duration of follow-up was 18.1 months for the 131 patients (range, 0.4−75 months). In all cases, 70 (53.4%) and 61 (46.6%) patients were stratified into low/low-intermediate risk and intermediate-high/high-risk, respectively, according to the IPI score. A total of 27 (38.6%) and 47 (77.0%) patients in the low/low-intermediate risk and intermediate-high/high-risk groups, respectively, had died.

**Table 1:** Clinical baseline characteristics of all PTCLs patients

Patient demographics	*N* = 131
Age, year	50.6 ± 18.3
Gender, *n* (%)	
Male	84 (64.1)
Female	47 (35.9)
Ann Arbor classification, *n* (%)	
I	16 (12.2)
II	9 (6.9)
III	39 (29.8)
IV	67 (51.1)
Stratification, *n* (%)	
Low/low-medium risk	70 (53.4)
High-medium/high risk	61 (46.6)
IPI score, *n* (%)	
0	16 (12.2)
1	24 (18.3)
2	30 (22.9)
3	35 (26.7)
4	22 (16.8)
5	4 (3.1)
B symptom, *n* (%)	51 (38.9)
Anthracycline regimen, *n* (%)	95 (72.5)
Biochemical test	
Hemoglobin, g/L	95.8 ± 24.0
White blood cell, 10^9^/L	3.8 (2.1, 6.4)
Platelets, 10^9^/L	131 (58, 191)
ESR, mm/h	29 (16, 61.5)
ALT, U/L	25 (12, 40)
AST, U/L	20 (14, 34)
Albumin, g/L	27.4 ± 5.8
ALP, U/L	87.5 (60.3, 131)
Creatine kinase, U/L	35.5 (23, 62)
Creatine kinase isoenzyme, U/L	11 (7.3, 16)
Blood urea nitrogen, mmol/L	7.5 ± 6.0
Blood creatinine, umol/L	77.6 ± 37.8
LDH, U/L	313 (234, 475)
EB virus, copies	50 (50, 892)
NT-pro BNP, pg/mL	539.4 (114.2, 3634)
d-Dimer, mg/L	3.3 (1.5, 9.5)
Troponin T, ng/mL	0.006 (0.003, 0.013)
ST-T changes, *n* (%)	17 (13.0)
Death, *n* (%)	74 (56.5)
Follow-up time, months	18.1 ± 15.7

Data are expressed as mean ± SD or median (interquartile range). Ann Arbor classification: clinical staging of malignant lymphoma. Stratification: low/low-medium risk (IPI/PIT 0–2), high-medium/high risk (IPI/PIT 3–5). IPI/PIT score system: IPI. B symptom: fever, night sweat, weight loss. ESR: erythrocyte sedimentation rate, ALT: alanine aminotransferase, AST: aspartate transaminase, ALP: alkaline phosphatase, NT-proBNP: N-terminal pro-brain natriuretic peptide.

### 3.2 Prognostic values of ST-T changes

**Figure 1:**
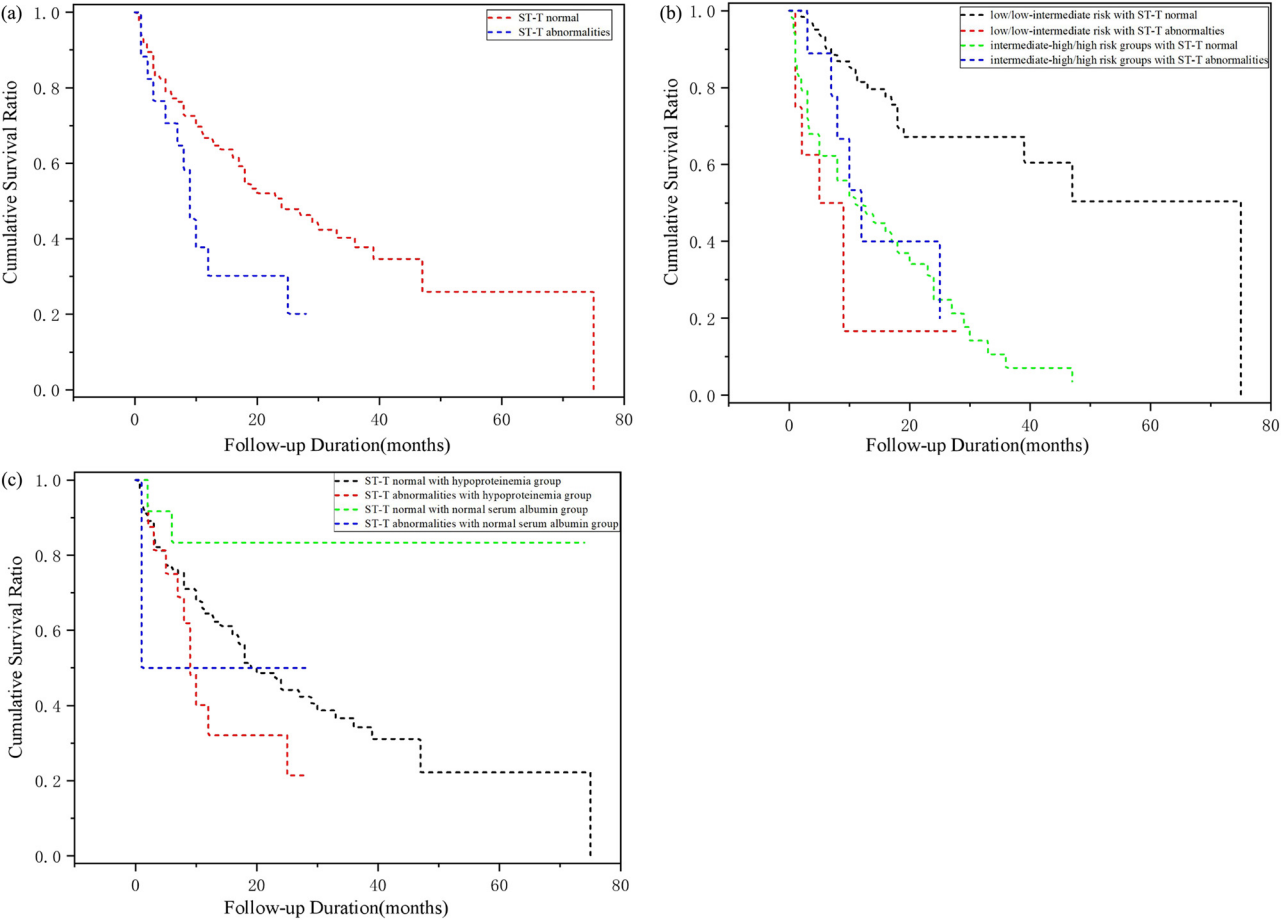
Survival curve analysis. (a) Kaplan–Meier survival curves according to the presence of ST-T normal and ST-T abnormalities, ST-T changes had worse cumulative survival ratio (*P* = 0.027). (b) Kaplan–Meier survival curves according to presence of ST-T normal and ST-T abnormalities and in the low/low-intermediate risk and intermediate-high/high risk groups, patients with ST-T segment abnormalities had a higher all-cause mortality than the patients with no definite abnormalities in both groups (*P* = 0.013; *P* ＜ 0.001). (c) Kaplan–Meier survival curves according to the presence of ST-T normal and ST-T abnormalities in the normal serum albumin and hypoproteinemia group, and patients with ST-T changes in the hypoproteinemia group showed a poor cumulative survival ratio (*P* = 0.021).

ST-T changes in ECG parameters were observed in 17 (13.0%) patients, and we found that all these patients had low or inverted T waves, among which 10 (7.6%) patients are accompanied by ST segment depression. ST-T changes are significantly associated with all-cause mortality (Figure 1a). The all-cause mortality was 70.6% (12/17) in patients with ST-T segment abnormalities and 54.4% (62/114) in patients with no definite abnormalities of the ST-T wave segments. Further, these ST-T changes remained independently correlated with all-cause mortality, irrespective of the stratification subtype, Ann Arbor classification, and serum albumin (HR = 2.042, 95% CI = 1.083−3.848, *P* = 0.027; Table 2).

**Table 2:** Univariable and multivariable analyses for overall mortality

	Univariable analysis			Multivariable analysis		
	HR	95% CI	*P* value	HR	95% CI	*P* value
Age	1.011	0.997–1.024	0.116			
Gender (female)	0.711	0.447–1.133	0.151			
Hemoglobin	0.983	0.974–0.992	<0.001	0.995	0.983–1.007	0.375
ESR	1.002	0.995–1.009	0.528			
EB virus	1.000	1.000	0.262			
Albumin	0.899	0.856–0.945	<0.001	0.919	0.872–0.969	0.002
LDH	1.000	1.000	0.002			
Creatine kinase	1.000	0.999–1.000	0.810			
Creatine kinase isoenzyme	0.996	0.976–1.016	0.679			
Blood urea nitrogen	1.049	1.015–1.084	0.005	1.007	0.996–1.049	0.756
Creatinine	1.006	1.001–1.012	0.029			
NT-pro BNP	1.000	1.000	0.046			
d-Dimer	1.013	1.007–1.019	<0.001			
Troponin T	7.275	0.143–369.648	0.322			
Ann Arbor classification						
I	1					
II	0.423	0.152–1.174	0.099			
III	0.431	0.204–0.912	0.028			
IV	0.392	0.191–0.806	0.011			
Stratification subtype						
Low/low-medium risk	1			1		
High-medium/high risk	3.507	2.159–5.697	<0.001	2.957	1.802–4.851	＜0.001
IPI/PIT score system						
0	1					
1	1.174	0.353–3.902	0.793			
2	2.114	0.693–6.453	0.189			
3	4.978	1.734–14.293	0.003			
4	5.780	1.934–17.275	0.002			
5	5.265	1.165–23.786	0.031			
ST-T changes	1.989	1.060–3.734	0.032	2.042	1.083–3.848	0.027

Ann Arbor Classification: Clinical staging of malignant lymphoma. Stratification: low/low-medium risk (IPI/PIT 0–2), high-medium/high risk (IPI/PIT 3–5). IPI/PIT score system: IPI. B symptom: fever, night sweat, weight loss. ESR: erythrocyte sedimentation rate, NT-proBNP: *N*-terminal pro-brain natriuretic peptide.

### 3.3 Prognostic values of ST-T changes in risk stratification

The average ages of patients in the low/low-intermediate risk and intermediate-high/high-risk groups were 46.7 ± 16.7 and 55.1 ± 19.1 years, respectively. Moreover, patients in the two groups accounted for 53.4% (70/131) and 46.6% (61/131) of all patients in this study. All-cause mortality was 38.6% (27/70) and 77.0% (47/61) for the two different risk stratification groups. In both groups, all-cause mortality was higher in the ST-T segment abnormalities group than that in the no definite abnormalities of the ST-T wave segments group (HR = 3.235, 95% CI = 1.283–8.159, *P* = 0.013; HR = 6.118, 95% CI = 2.410–15.534, *P* < 0.001; Figure 1b).

### 3.4 Prognostic values of ST-T changes in PTCLs with hypoproteinemia

We further explored the interactions between the ST-T changes in PTCLs patients with hypoproteinemia and all-cause mortality in multivariable Cox proportional hazard regression models. The mean albumin level was 27.4 ± 5.8 g/dL. Among 131 patients, 117 patients (89.3%) patients presented hypoproteinemia, while 14 (10.7%) patients had normal serum albumin levels. ST-T changes were observed in 1 patient among 14 patients with normal serum levels (7.1%) and in 16 patients out of 117 patients with hypoproteinemia (13.7%). Significantly, patients with ST-T changes and hypoproteinemia showed a poor cumulative survival ratio (HR = 5.930, 95% CI = 1.310–26.836, *P* = 0.021). However, no significant prognostic potential of ST-T changes was found between patients with normal ST-T segments and ST-T abnormalities in the normal serum albumin group (HR = 3.927, 95% CI = 0.355–43.434, *P* = 0.265; Figure 1c).

## 4 Discussion

ST-T changes are useful predictors of survival in various malignancies such as gastrointestinal tumors and breast cancer [16,17]. However, there is little evidence regarding the connection between ST-T changes and PTCLs. In this study, we explored the relationship between ST-T changes and all-cause mortality in PTCLs patients and identified ST-T changes as independent predictors for all-cause mortality in PTCL patients.

PTCLs often have poor clinical outcomes due to their heterogeneity and aggressive nature. Anthracycline-based chemotherapy remains the most commonly used frontline regimen for PTCLs. It is estimated that the 2-year mortality rate of cancer patients diagnosed with anthracycline-induced cardiotoxicity is approximately 50% [22]. The underlying mechanism is believed to be related to excessive reactive oxygen species production, intracellular calcium dysregulation, cytokine release mediated by activation of the innate immune system, high affinity for myocardial phospholipids, and mitochondrial functional disturbances [9,23]. Most antitumor agents can induce cardiotoxicity and affect patient prognosis. The current prognostic factors of PTCLs commonly rely on age, clinical stage, classification, Epstein–Barr (EB) virus quantitative, serum LDH, and specific cytogenetic abnormalities. Although there are many factors affecting prognosis, the overall survival (OS) rate of PTCLs in 10–15 years is 10% [4]. Therefore, early and prompt recognition of abnormal electrical activities and timely diagnostic evaluation of cardiac burden are essential when choosing suitable therapies and improving patient prognosis.

ECG can be used to measure the electrical activity of the heart. Further, ECG can be utilized to reflect changes in heart physiological activity and correctly identify different cardiac events, including ST-T changes that may indicate a potential risk of arrhythmias and sudden cardiac death. Studies have shown that ST-T changes are closely affiliated with unfavorable prognosis [24,25]. In our study, the two specific ST-T changes of ischemic T-wave level or inversion and ischemic ST-segment depression all indicate endocardial myocardial injury, and ST-T changes were found to be independently related to all-cause mortality in PTCLs. Except for other secondary inflammations, ST-T changes are reliable prognostic indicators of myocardial ischemia [26,27]. Interestingly, we found that all-cause mortality was higher in patients with ST-T abnormalities than in those patients with no definite abnormalities of the ST-T wave segments in all risk stratifications (*P* < 0.05). The results indicate that a progressive delay in cardiac repolarization and myocardial ischemia develop in PTCLs and ST-T changes are useful factors for analyzing the prognostic outcomes in PTCLs patients.

Albumin is a significant predictor of survival in patients with diffuse large B-cell lymphoma [28]. Moreover, it is an independent prognostic agent of OS in PTCL patients [29]. We evaluated several related prognostic factors in both groups that may affect survival in this research, and albumin was considered an independent risk factor based on the multivariable Cox regression analysis. Further, significant differences in all-cause mortality could be found between patients with ST-T changes and normal ST-T segments within the hypoproteinemia group (*P* = 0.021). The suspected reasons for hypoproteinemia are impaired liver function, malnutrition, and high globulin levels [28]. Therefore, ST-T changes are suggested to have a statistically significant impact on all-cause mortality in PTCL patients with hypoproteinemia.

The atypical ST-T segment in ECG parameters represents myocardial repolarization abnormalities. The most frequently used anthracycline chemotherapeutic drugs that influence ventricular repolarization in PTCL patients can elicit ST-T changes in the surface ECG. As it may enable early detection of cardiotoxicity, clinical ambulatory electrocardiogram monitoring is warranted during PTCL treatment. Twelve-lead ECGs are easily available and relatively inexpensive for prognostic use in patients with PTCLs.

The present study had several strengths and limitations. This is the first study focusing on the potential prognostic role of ST-T changes on all-cause mortality in PTCL patients. As our data are based on a large general hospital in Northwest China, our findings can be generalized to a certain extent. However, this was a single-center retrospective study, and data on echocardiography are lacking. In addition, the sample size was relatively small in the stratified analysis. Moreover, we have not further analyzed and compared different types of ST-T changes and the differences between anthracycline- and nonanthracycline-induced ST-T changes due to the relatively small sample size and the low proportion of ST-T changes.

## 5 Conclusion

We explored the clinical and prognostic value of ST-T changes in PTCL patients and found that ST-T changes led to higher all-cause mortality, especially in the intermediate-high/high-risk and hypoproteinemia groups. Thus, ST-T changes may be markers of all-cause mortality to provide prognostic information for PTCLs.

## Abbreviations

CI: confidence interval
ECG: electrocardiogram
ECOG: Eastern Cooperative Oncology Group
HR: hazard ratio
IPI: International Prognostic Index
LDH: lactate dehydrogenase
NCCN: National Comprehensive Cancer Network
OS: overall survival
PTCLs: peripheral T-cell lymphomas
ROS: reactive oxygen species
